# Midwives’ perspectives of respectful maternity care during childbirth: A qualitative study

**DOI:** 10.1371/journal.pone.0229941

**Published:** 2020-03-09

**Authors:** Maryam Moridi, Farzaneh Pazandeh, Sepideh Hajian, Barbara Potrata

**Affiliations:** 1 Student Research Committee, Department of Midwifery and Reproductive Health, School of Nursing and Midwifery, Shahid Beheshti University of Medical Sciences, Tehran, Iran; 2 Midwifery and Reproductive Health Research Center, School of Nursing and Midwifery, Shahid Beheshti University of Medical Sciences, Tehran, Iran; 3 Independent scholar, Rotterdam, The Netherlands; Federal University of Sergipe, BRAZIL

## Abstract

The adoption of respectful maternity care during labor and birth is a complex process which needs both scientific and interpersonal skills of providers. In this regard, identifying the potential barriers and applying effective strategies for implementing respectful maternity care are essential. This study aimed to explore the perceptions of Iranian midwives regarding respectful maternity care during labor and childbirth. This was a qualitative study which was conducted from September-December 2018 in two non-teaching public hospitals in Tehran, Iran. Twenty-four semi-structured interviews were conducted with midwives, who had more than one year work experience in labor and childbirth units, through a purposive sampling method. A content analysis approach was used to analyze the data and identify themes. Three themes were extracted including “showing empathy”, “women-centered care” and “protecting rights”. Showing empathy reflects that “establishing a friendly relationship” and “being with women”. Women-centered care encompassed “keeping women safe” and “participating in decision making”. Protecting rights reflected a need for “safeguarding dignity” as well as “giving equal care” and “preparing appropriate environment”. Iranian midwives considered respectful maternity care a broader concept than just preventing mistreatment. Providing supportive care through friendly interaction with women was the first step for providing respectful maternity care. Respectful care is also promoted by providing safe care, implementing evidence-based care and involving women in their care as well as by providing an appropriate environment for women, families and caregivers.

## Introduction

Childbirth is an important event in women’s life and all women need and deserve to receive respectful care during labor and childbirth [1]. Recommendations from the World Health Organization (WHO) in 2018 emphasized the quality of interaction between women and their health care providers and considered good interactions as a prerequisite for positive outcomes of childbirth [2]. This interaction involves health care professionals’ preserving women’s respect and providing essential information and emotional support during labor and birth [3–5]. Respectful Maternity Care (RMC) has been recognized as an essential strategy for improving quality and utilization of maternity care [6]. It is defined as a universal human right that encompasses the principles of ethics and respect for women’s feelings, dignity, choices and preferences [7]. Indeed, RMC is an approach to care which emphasizes the fundamental rights of women, newborns, and families, and that enhances adequate access to evidence-based care while recognizing the unique needs and preferences of both women and newborns [8]. The White Ribbon Alliance (WRA) has defined seven domains of RMC during childbirth using a rights-based approach including physical abuse, non-consented care, non-confidential care, non-dignified care, discrimination, abandonment of care, and detention in facilities [1], however, operational components of RMC in terms of specific behaviors, practices, or standards in research and program implementation are often variable [8].

There are several studies throughout the world that attempted to explore the components of RMC during labor and birth from the laboring women’s and providers’ perspectives. These components generally include providing safe and timely care, nurturing positive interactions between midwives and women, protecting confidentiality, maintaining an active role in the labor process, obtaining the women’s consent before performing procedures, providing information regarding procedures to women, respecting patient privacy, and promoting freedom of choice as it related to position for labor and birth [8–12]. From the laboring women’s perspective, an essential component in the improving quality of care and birth satisfaction is providers’ respectful behavior. This is becoming a critical indicator of maternal health care [13].

Iran has good maternal health indicators and has reached the targets of the fifth Millennium Development Goal (MDG), that of improving maternal health [14]. Around one million women give birth each year in Iran, with 96% of births taking place in health facilities and 97% managed by skilled attendants [15]. While respecting laboring women has been noted in Iran’s National Guidelines for Normal Childbirth, the strategies and indicators to implement RMC have not been addressed clearly [16]. Despite the adoption of a Mothers’ Bill of Rights in 2003 [17], a study from Iran reported that the quality of childbirth care is not desirable including support groups, timely attention, rapid prevention and detection, service continuity, respect, safety, access, and basic facilities [18]. The purpose of this qualitative study was to provide insight into Iranian midwives’ perspectives on RMC during labor and childbirth in non-teaching hospitals in Tehran.

## Methods

### Study design

This qualitative study, using conventional content analysis approach, was conducted from September-December 2018 in two non-teaching public hospitals in Tehran, Iran.

### Setting and participants

The midwives were recruited from two non-teaching public hospitals in Tehran, Iran, from September to December 2018, by using purposeful sampling method. The inclusion criteria were: 1) at least a bachelor’s degree of midwifery, 2) work experience for one year or more in labor and birth units, and 3) having complete responsibility of labor and childbirth care. Iranian midwives receive four years of training in an academic setting and in the past could practice independently in prenatal, labor, childbirth and postpartum care [19]. However Iranian midwives today have little authority in providing childbirth care and for the most part the perinatal care process is managed by obstetricians in teaching hospitals. In some non-teaching public hospitals, childbirth care is performed by midwives under the obstetricians’ supervision [20].

### Data collection

Participants were interviewed at a time and place of their convenience. Semi-structured, in-depth, face to face interviews were conducted by the first author. First three pilot in-depth interviews were conducted with midwives who were screened against inclusion criteria and worked in the study hospitals. The interviews were conducted following a semi-structured interview guide which was developed through literature reviews and expert panel (midwifery, nursing and ethics academics, and heads of birth units). The interview began with a question about midwives’ personal perception of respectful care for laboring women and continued with more general questions on provision of respectful care during childbirth. Then interviews continued with follow-up probes including: Would you please explain it more?; how it would be done; would you please tell me an example about this? The duration of the interview sessions varied from 60 to 120 min and the mean duration of interviews was 80 min. All interviews were audio-recorded with participants’ permission and transcribed verbatim in the Persian language prior to the next interview.

A qualitative content analysis was employed to explore Iranian midwives’ perceptions about RMC. Data analysis was undertaken concurrently with data collection, following Graneheim and Lundman’s (2004) method, including transcription of the whole interview immediately after each interview, reading the entire transcription of the interview to achieve an overall understanding of its content, specifying basic codes, classifying initial similar codes in more comprehensive sub-themes, and ultimately, extracting themes from the sub-themes [21]. The first three interviews were coded by each researcher of the study independently, after which the codes were compared. If the codes differed, the disagreement was resolved by consensus. The first three interviews were entered into MAXQDA and then other interviews were added and analyzed. Data saturation was achieved at 24 interviews.

Trustworthiness of the data followed strategies proposed by Lincoln and Guba in 1994 as cited in Polite & Beck, 2010 [22]. All interviews were carried out by MM who was trained in conducting qualitative research and interviewing techniques. The interviewer had a prolonged engagement with data and the process of data collection and analysis which lasted about two years. Finally, the sufficient numbers of interviews were performed to ensure the saturation of concepts. At the end of the study the methods of member- and peer- checking were used. The printed transcribed files were returned to participants for their review of accuracy. Additionally, peer checking was conducted through sending the transcripts, codes and themes to four experienced qualitative researchers in reproductive health and social sciences to review the credibility of the extracted themes and sub-themes. Finally, in addition to audio-records and transcripts, multiple data sources, including field notes and observation were used.

### Ethics

This study was a part of doctoral dissertation which was approved by the Ethics Committee of the Research Deputy at Shahid Beheshti University of Medical Sciences in Tehran (approval code: IR.SBMU.PHNM.1396.810). The permission for performing the study was taken from the selected hospitals. Midwives were informed about the research objective and participant’s freedom to join or leave the interview. They were also assured of confidentiality of information. Only those midwives who agreed to participate and signed the written consent letter were interviewed.

## Results

The age range of midwives was 24–55 years. Majority of participants hold only BSc degree and were staff midwives (Table 1).

**Table 1:** The demographic characteristics of midwives in two non-teaching public hospitals of Tehran, Iran.

**Age (year) (Range)**	24–55
**Age (Year) (Mean)**	38.5
**Education** (Number)	
BSc	13
MSc	6
PhD	5
**Work experience (Year) (Range)**	1–29
**Work experience (Year) (Mean)**	15
**Position (Number)**	
Head of midwifery or Midwife in charge	2
Staff midwife	17
Midwifery instructor	5

The analysis of in-depth interviews finally led to the generation of three themes and seven sub-themes which are listed in Table 2 and are described below.

**Table 2:** The main themes and sub-themes extracted from Iranian midwives’ perception regarding respectful maternity care during labor and childbirth.

The number of instances each sub theme came up during the interviews (n = 24)	Sub-themes	Themes
24	Establishing friendly relationship	Showing empathy
22	Being with women	
18	Keeping women safe	Women-centered care
8	Participating in decision making	
20	Safeguarding dignity	Protecting rights
15	Giving equal care	
5	Preparing appropriate environment	

Three themes were derived from the midwives’ perceptions of RMC during labor and birth, including showing empathy, women-centered care, and protecting rights. Showing empathy reflected the need for building trust and confidence in women. Women-centered care described the importance of providing the best care, and prevention of any complication as well as the importance of involving women in their care by providing adequate information and taking informed consent on care. Protecting rights also reflected a need to protect the dignity of the women through respect for their privacy, confidentiality, equality, and the provision of suitable environment.

### Theme 1: Showing empathy

Midwives take the first step in providing optimal care for women. Showing empathy had two aspects, establishing friendly relationship and being with women.

#### Establishing friendly relationship

The majority of midwives, especially those with longer work experience, believed that establishing a good and friendly relationship was essential for providing RMC. The midwives stated that pregnant women are worried about their own and their baby’s health status and believed that creating a good relationship would make very friendly atmosphere to preserve RMC. A midwife said:

“*Respectful care is that we have a good relationship with women*. *We need to communicate in a way that they can trust us*”.

(Midwife 7, 42yrs, 17yrs work experience, MSc in midwifery)

Midwives stated that using kind words and respectful language when talking to women were effective measures for establishing a friendly relationship.

“*When a mother arrives*, *we should kindly welcome her and call her name in a respectful manner*. *In this way*, *she would feel comfortable*”.

(Midwife 13, 35yrs, 12yrs of working experience, MSc in midwifery)

#### Being with women

Some midwives believed that one of the most important parts of respectful care is to understand the unique situation of the laboring women and their families. A midwife stated:

“*I should understand the woman’s situations*. *If we want to perform vaginal examination*, *and the woman has labor pain*, *it is better to wait until she gets calm*”.

(Midwife 2, 24 yrs old, 2 yrs of working experience, BSc in midwifery)

In the study hospitals, the women were mainly not allowed to have a companion of their choice. Midwives believed that the presence of companion is another important way to provide respectful care for women.

“*One of the best supports that we can provide for woman is to allow companion of her choice to stay with her*. *If the companion has taken part in the birth-preparation classes*, *she/he can help the mother to have a better experience*.”

(*Midwife 20*, *39 yrs old*, *16 yrs of working experience*, *BSc in midwifery*)

### Theme 2: Women-centered care

The women-centered care includes two sub-themes: keeping women safe that refers to the provision of evidence-based and harm-free care for women. It also includes participating in decision making that discusses the need of providing adequate information about procedures and interventions and involving women in decision-making about their care.

#### Keeping women safe

The participant midwives believed that science and ethics are interconnected. In the study hospitals, childbirth care was medicalized and unnecessary interventions such as accelerating and augmenting labor using oxytocin and performing episiotomy were used for the low risk women. The midwives believed that unnecessary interventions without medical indication should be eliminated and any harm to mothers and babies should be prevented. One midwifery lecturer stated:

“*First of all*, *we all want to give a scientific care and perform it accurately*. *We don’t need to do some interventions such as unnecessary vaginal examinations*, *forced early admission and electronic fetal heart monitoring continuously during labor and birth*.”

(Midwife 17, 40 yrs, 10 yrs work experience, PhD in Midwifery)

Some midwives stated that timely presence at bedside help to keep women’s health and form the important part of respectful care.

“*When a woman needs a specific care*, *we should provide it for her as soon as possible*, *because delay in taking care would have undesirable consequences for both mother and child*. *In this way I think the women’s respect will be protected*.”

(Midwife 12, 40 yrs old, 8 yrs of working experience, PhD in reproductive health)

A midwife in charge of the labor unit stated that it is difficult to provide women-centered and respectful care when the births are managed by obstetricians. She emphasized that the midwife-led care is an appropriate model of care for improving the RMC.

“*Disrespect is consequence of working in a medicalized context*. *They (obstetricians) treat laboring women hastily and completely medically*. *If we had midwife-led birth centers*, *then we could provide respectful care for women*”.

(Midwife 4, 46 yrs old, 25 yrs of working experience, BSc of midwifery)

#### Participating in decision making

Most midwives considered involving women in the care process and decision making as an essential part of the RMC. This would contribute to women-centered care by recognizing women’s preferences and expectations. Midwives believed that women need to get information about progress of labor and these information could help them to participate in decision making about their cares and interventions. One midwife said:

“*We respect the women when give information about progress of labor*. *We should tell them what is going on at every stage and what they can do to help themselves*, *this would reduce their stress*. *We should introduce caregivers and make them familiar with rooms and equipment as well*.”

(Midwife 9, 46 yrs old, 24 yrs of working experience, BSc in midwifery)

Midwives expressed that women also need to be informed about the care and interventions that would be performed. They believed that access to information is needed for the active participation of women in the process of care. In this regard, a midwife said:

“*If we want to check the fetal heart rate or doing an episiotomy*, *we should first explain the procedure and the necessity of performing of that*, *then if woman agrees*, *it could be done*”.

(Midwife 8, 38 yrs, 22 yrs of working experience, BSc of midwifery)

### Theme 3: Protecting rights

This theme referred to the importance of providing a caring environment in which the privacy, equality, non-discrimination and comfort was provided. This theme includes three sub-themes of safeguarding dignity, giving equal care and preparing appropriate environment.

#### Safeguarding dignity

Some midwives stated that preserving dignity is to preventing any mistreatments with laboring women. They emphasized that mistreatments that mainly were performed by obstetricians and young midwives, should be eliminated.

“*Some less experienced midwives do not know how to deal with a laboring woman when she is in pain and shouting*. *They may not treat women properly and show unpleasant reactions*. *Women’s dignity should be protected by preventing provider’s disrespectful behaviors*.”

(Midwife 11, 50 yrs old, 25 yrs of working experience, BSc in midwifery)

The Iranian midwives considered preserving women’s privacy during labor and childbirth in frequently overcrowded units as an important part of RMC.

“*Preserving privacy is an important issue for the women in labor*. *Some midwives think that due to labor pain and existence of only the female staff in the units*, *it is not necessary to pay attention to women’s privacy*. *But I believe that if the women’s privacy is not preserved*, *they may be shameful and feel disrespect*.”

(Midwife 21, 45yrs, 20 yrs of working experience, BSc in midwifery)

The majority of participants stated that a significant example of assuring women’s privacy is covering their bodies with a bed sheet during examinations.

“*Dignity means to preserve the privacy of women*. *When I start Fetal Heath Monitoring (FHM) for a woman*, *the first thing that I consider is to cover her body using a bed sheet*”.

(Midwife 8, 48 yrs old, 22 yrs of working experience, BSc in midwifery)

Midwives also mentioned that women should not be examined in attendance of unnecessary individuals.

“*It is important when we perform any intervention for women*, *nobody should be a there*”.

(Midwife 13, 35yrs, 12 yrs of working experience, MSc in midwifery)

#### Giving equal care

Some midwives stated that caregivers should respect everyone and avoid discrimination on grounds of women’s culture, customs and religion. Such attitude facilitates and improves the implementation of respectful care. In this regard, a midwife said:

“*When they come here*, *the rights of women from every culture and traditions should be protected and we must pay attention to them*”.

(Midwife 15, 39 yrs old, 15 yrs of working experience, MSc in midwifery)

The midwives, participating in this study also believed that having prior knowledge of different cultures and taking into account cultural and religious differences help in providing equal and fair care for the women.

“*When the women are trying hard to push*, *I ask them to rely on the God and pray by reading Doa (written praying)*. *Sometimes I think*, *maybe this is not believed by all*, *or some people who are from other countries might have different beliefs*.”

(Midwife 18, 30 yrs old, 9 yrs of working experience, BSc in midwifery)

#### Preparing appropriate environment

Midwives believed that providing a comfortable, clean, and quiet labor and birth environment with sufficient equipment for laboring women was essential for providing respectful care. They stated that these services give women a sense of security and comfort.

“*When a woman enters the birth unit and sees untidy environment*, *she may get stressed and feels some sort of disrespect; we should try to provide a convenient setting in which women could be relaxed*”.

(Midwife 11, 50 yrs old, 25 yrs of working experience, BSc in midwifery)

One of the desirable recent changes in the labor unit of hospitals is establishing the Labor and Delivery Room (LDR), which improves the privacy of the women. Midwives believed that LDR provide the comfort for women and ultimately maintained their dignity.

“*Some of units now have LDR which are very comfortable place for women*. *As women stays in a single room that really preserves the privacy of the women*”.

(Midwife 14, 38 yrs old, 12 yrs of working experience, BSc in midwifery)

In the study hospitals, companions stayed outside and even in the labor unit did not have an appropriate place for waiting. A number of midwives also stated that companions of women should also have a suitable waiting room with adequate seats.

“*The companions stay outside in a cold place*, *from night until morning*. *They lose their patience*, *so they may get easily nervous*. *If they could stay in a suitable place*, *they would be comfortable and cooperate with us to support the women*.”

(Midwife 24, 42 yrs old, 19 yrs of working experience, BSc in midwifery)

## Discussion

This study presented the perception of Iranian midwives about RMC during labor and childbirth and demonstrates that these midwives believe that RMC is more than merely preserving women’s dignity while giving birth. Participating midwives defined RMC as showing empathy, providing women-centered care and protecting rights. Several studies that investigated the childbearing women’s experiences supported the accuracy of the perceptions of the midwives [8,23,24]. They indicated that the women’s experiences are multidimensional and women did not want to be treated as the medical patients during labor and childbirth, but rather as a human beings with feelings, expectations [8,23,25]. A study from Iran showed that women’s needs and expectations fell into seven main categories: Physiological, psychological, informational, social and relational, esteem, security and medical needs [24].

Our study indicated that the important aspect of RMC is to show empathy to the woman by being with women and establishing a friendly relationship. In fact, the midwives believed that providing a supportive and intimate interaction with women was the key determinant of RMC. The establishing of warm and friendly relationships with women is reported in several studies from the UK, Norway, Sweden, New Zealand and Australia [12, 26–29]. Bradfield and his associates also defined the term of “being with women” as the fundamental component of the midwifery profession and philosophy [12]. Nigerian and Ugandan women’s experiences also indicate the positive emotional and interpersonal experiences of care have equal value with the clinical and contextual environmental factors [28,30].

Another feature of RMC emphasized by the midwives in our study was providing timely, and up to date care that includes adequate information for a woman to participate in decision-making. Furthermore, a midwife highlighted the importance of birth centers where midwifery model of care would be provided for women and could be a facilitator for promoting RMC. Providing safe, efficient, effective, and timely care by taking informed consent has been reported in several studies [8–10]. In addition, some studies concluded that birth centers create a positive birth experiences [31,32].

Iranian midwives’ narratives indicated that the RMC referred to respect to the women’s customs, religion, ethnicity, autonomy, privacy. A systematic review demonstrated that women’s experiences of childbirth are shaped by culture. Providing culturally competent maternal health care will improve the quality of the birth experiences for women and their families [33]. Behruzi et al. (2014) highlighted the importance of respecting the women’s cultures, values and beliefs from the midwives’ views [34]. Also, Aziato et al, (2016) stated that, in all spheres of midwifery, spirituality should be considered as an integral part of the care provided to women and their families [35].

This qualitative study was a first study in its kind which was conducted in the context of middle income countries in the Middle East. The interview with adequate number of midwives is strength of this study. This study was conducted in two public hospitals in Tehran but the results may be applied to other provinces in Iran as well as similar contexts and cultures in other low- and middle income countries.

### Limitation of the study

This study was carried out in two non-teaching hospitals in the South of Tehran, therefore this may not reflect the perceptions of midwives in other parts of Iran. The participant midwives in the study hospitals provided labor and childbirth care under supervision of obstetricians. Consequently, midwives’ perception who work in teaching hospitals where obstetric residents manage all vaginal births and midwives are less involved in normal childbirth, may be different.

## Conclusion

Midwives’ perceptions indicate that RMC was broader concept rather than preventing disrespect and preserving women’s rights and dignity. This research, along with all the articles surveyed, seem to make the same point about what women want in labor kindness, respect and inclusion, safety. It is important to maintain appropriate interpersonal communication between caregivers and women during labor and childbirth. Promoting RMC by implementing women-centered, evidence-based and humanized care is recommended. The pre-service and in-service midwifery trainings, improvement of environmental conditions, and streamlining of maternity systems by close collaborations of health providers are needed. These should be considered by policy-makers to design culturally appropriate interventional program to promote RMC. We seem to have a global consensus; now we as a childbirth care community need to decide what to do next. Further testable research to confirm these results is required.
